# An assessment of the performance of the national health research system in Mauritius

**DOI:** 10.1186/s12913-023-09208-x

**Published:** 2023-03-06

**Authors:** Laurent Musango, Ajoy Nundoochan, Yogendranath Ramful, Joses Muthuri Kirigia

**Affiliations:** 1 World Health Organization, Country Office, Antananarivo, Madagascar; 2 World Health Organization, Country Office, Port Louis, Mauritius; 3 Independent Consultant (Previously Ministry of Health and Wellness), Port Louis, Mauritius; 4 African Sustainable Development Research Consortium (ASDRC), Nairobi, Kenya

**Keywords:** Barometer, National health research system, Research for health, Leadership, Governance, Financing

## Abstract

**Background:**

The goal of universal health coverage (UHC) is that every individual has access to high-quality health services without running the consequences of financial hardship. The World health report 2013 "Research for universal health coverage" states a performant National Health Research Systems (NHRS) can contribute by providing solutions to challenges encountered in advancing towards UHC by 2030. Pang et al. define a NHRS as the people, institutions, and activities whose primary aim is to generate and promote utilization of high-quality knowledge that can be used to promote, restore, and/or maintain the health status of populations. The WHO Regional Committee for Africa (RC) adopted a resolution in 2015 urging member states to strengthen their NHRS to facilitate production and utilization of evidence in policy development, planning, product development, innovation, and decision-making. This study aimed to calculate NHRS barometer scores for Mauritius in 2020, identify the gaps in NHRS performance, and recommend interventions for boosting the Mauritius NHRS in the pursuit of UHC.

**Methods:**

The study used a cross-sectional survey design. A semi-structured NHRS questionnaire was administered and complemented with a review of documents archived in pertinent Mauritius Government Ministries, universities, research-oriented departments, and non-governmental organizations websites. The African NHRS barometer developed in 2016 for countries to monitor the RC resolution implementation was applied. The barometer consists of four NHRS functions (leadership and governance, developing and sustaining resources, producing and utilizing research, financing research for health [R4H]), and 17 sub-functions, e.g., existence of a national policy on research for health (R4H), presence of a Mauritius Research and Innovation Council (MRIC), existence of knowledge translation platform.

**Results:**

In 2020, Mauritius had an overall average NHRS barometer score of 60.84%. The four NHRS functions average indices were 50.0% for leadership and governance, 77.0% for developing and sustaining resources, 52.0% for producing and utilizing R4H, and 58.2% for financing R4H.

**Conclusion:**

The performance of NHRS could be improved through the development of a national R4H policy, strategic plan, prioritized agenda, and national multi-stakeholder health research management forum. Furthermore, increased funding for the NHRS may nurture the human resources for health research capacities, hence the number of pertinent publications and health innovations.

## Background

At the core 2030 Agenda for Sustainable Development is the goal of Universal Health Coverage (UHC) with every individual and community, irrespective of their circumstances, receiving the health services they need without risking financial hardship. Mauritius has been implementing various health programmes to accelerate the attainment of United Nations Sustainable Goal 3 (SDG3) to “Ensure healthy lives and promote well-being for all at all ages”. The SDG concerned are 3.1.1 (Maternal Mortality ratio), 3.2.1(Under 5 Mortality rate), 3.2.2 (Neonatal mortality rate) [1–3]. Progress towards the goal of UHC in the post-2015 sustainable development agenda will be difficult for African countries without strengthening of their national health research systems (NHRS) to yield knowledge on health determinants and evidence to inform health policy makers decisions. Mauritius is no exception and will benefit accordingly.

The present study is informed by the World health report 2013 "Research for universal health coverage"(2013) and three core guiding messages. First, an effective NHRS has the potential to generate evidence and guide policy makers on the most effective solutions in ensuring the core fundamentals of UHC. These are namely access to high-quality services for health promotion, prevention, treatment, rehabilitation, palliation, and financial risk protection. Second, mindful that the focus should not only be towards research production but also translating the findings into action, health systems research should be the responsibility of public health programmes which have better insights of the demand and supply for health services. Third, the world health report posits that an effective research system would ensure national priority research agenda is well planned and developed, adequately funded, and capacitated to guarantee appropriate use of findings and evidence generated [4].

Moving forward, the World Health Organization (WHO) Regional Committee for Africa (RC) adopted resolution AFR/RC65/R2 entitled "Research for Health: A Strategy for the African Region, 2016–2025". The Resolution urged the Member States to strengthen their NHRS to stimulate knowledge production and use to promote, restore, and maintain population health. It also requested WHO and the Member States to conduct at every two years a mapping of the status of NHRS [5]. Notwithstanding, the growing renewed interest for health system research, globally a dearth of actions undertaken to develop national priority research agenda with a view to advancing UHC remains a concern [6, 7].

According to the Commission on Health Research for Development, research for health (R4H) has immense power to reduce premature deaths, morbidity, and disability[8]. Efficient conduct of R4H to buttress pursuit of nationally and internationally agreed on health development goals requires a functional NHRS. In their pioneering study, on the development of a conceptual framework for NHRS, Pang et al*.* [9] defined a NHRS as.

“*the people, institutions, and activities whose primary purpose in relation to research is to generate high-quality knowledge that can be used to promote, restore, and/or maintain the health status of populations; it should include the mechanisms adopted to encourage the utilization of research*” (p.816).

Their framework consists of four functions, that is, leadership and governance, developing and sustaining resources, producing, and utilising research, financing R4H. Since then, the framework has been used to describe and analyse overall NHRS in Africa [10–16], Americas [17–20], as well as in Asia, Eastern Mediterranean, Europe, and Western Pacific. However, none of the studies referred above developed indices for tracking the performance of NHRS.

In 2016, Kirigia et al*.* [21] designed the African NHRS barometer with four functions and 17 sub-functions and used the 47 World Health Organization (WHO) African Region countries data gathered in 2014 to operationalise it. Senkubuge et al*.* [22] used 2018 survey data to estimate NHRS barometer scores for South Africa, identify gaps in sub-functions, and make contextualised recommendations for enhancing its performance.

In 2018, the WHO Regional Office for Africa, the European and Developing Countries Clinical Trials Programme (EDCTP), and the Tackling Infections to Benefit Africa collaboratively, used the African NHRS barometer [7] to monitor the progress made by the 47 countries of the African Region in strengthening the NHRSs compared to the 2014 baseline [15, 23].

The NHRS barometer score for Mauritius improved substantially from 19% in 2014 [23] to 44% in 2018 [7]. It is necessary to assess and compare the progress made in strengthening the Mauritius NHRS between the last assessment in 2018 and 2020. For example, the EDCTP NHRS 2020 survey among its 17 African Participating States (Angola, Burkina Faso, Cameroon, Congo, Ethiopia, Gabon, The Gambia, Ghana, Mali, Mozambique, Niger, Nigeria, Senegal, South Africa, Tanzania, Uganda, Zambia) revealed an improvement in the average overall NHRS barometer score from 62% in 2018 to 65% in 2020[24].

Most of the health-related SDG targets are either achieved or on track, except for one not achieved. The interventions to address the unfinished agenda will be on multisectoral activities and focusing on health promotion. The myriad and wide array of interventions required emphasise the importance of monitoring and strengthening NRHS performance to ensure generation of knowledge to address local health challenges and progress towards UHC. Evidence generated will unravel the design of affordable service delivery models and strategies to optimise the use of existing solutions to address health systems challenges preventing achievement of SDG Target 3.8 of UHC, eliminate inequities to access health services and pursue the objective of UHC.

Over an almost similar 4 years period interval as the NHRS barometer score, UHC service coverage index (UHC SCI) to monitor SDG indicator 3.8.1 ( presented on a scale of 0 to 100 and computed around 14 tracer indicators extracted from various sources and organized into four broad categories of service coverage, namely reproductive, maternal, newborn and child health, infectious diseases, noncommunicable diseases, and service capacity and access) has improved from 63% in 2015 to 65% in 2019 [25, 26].

Healthcare within the public sector in Mauritius is delivered through a three-tier system. At the tertiary care level, specialised hospitals (5) and medical centres (2) function as the last referral centres. The secondary care level comprising two district hospitals and five regional hospitals provides primary inpatient and outpatient medical care for an extensive decentralised network of primary healthcare facilities. The latter comprises of community hospitals (2), mediclinics (7), area health centres (19) and community health centres (114). All health services provided at the three levels (primary, secondary and tertiary) are available free of charge. Conversely, all health services in private health facilities are available against payment. Private healthcare has evolved in two forms: private practice of medical and dental care practitioners, and private clinics with inpatient beds and facilities for examination, consultation and diagnostic procedures. There are 13 clinics with inpatient and outpatient services operating in the private sector. In addition, there are six dialysis clinics, including those in private clinics [27].

General government health expenditure (GGHE) accounts for approximately 2.85% of gross domestic product (GDP) and 10% of general government expenditure. Notwithstanding free access to healthcare in public sector, private health expenditure accounted for 53.8% of Total Health Expenditure. Out of pocket (OOP) expenditure on health and voluntary insurance reimbursements, accounted for 83% and 13%, of private health expenditure, respectively. The trend between GGHE and Household OOP Expenditure on health has reversed and since 2017 GGHE has surpassed the Household OOP Expenditure [28]. The incidence of catastrophic health expenditure due to OOP (at 10% of household total income) increased from 6.5% in 2006 to 8.85% in 2012 and dropped slightly to 8.2% in 2017 [29, 30].The persons whose OOP on health care exceed the threshold of 10% are at risk of a financial catastrophe and impoverishment [31]. The sub-optimal health-related indicators call for scientific research on the situation (burden and determinants), improvement in coverage and effectiveness of existing interventions, and feasibility of developing new capacities (tools and products). These relate to noncommunicable diseases (NCD), communicable, maternal, neonatal, and nutritional diseases (CMNND), injuries, national health system and other systems that address social determinants of health (SDH) [32].

The specific objectives of this study were to calculate NHRS barometer score for Mauritius in 2020, identify the gaps in NHRS performance, and recommend interventions for boosting the Mauritius NHRS production of pertinent evidence and promotion of its utilization to effective coverage of essential health services coverage, that is, UHC SCI components.

## Methods

### Steps in developing and estimating the Mauritius NHRS barometer

The process of estimating Mauritius NHRS barometer replicates the methodology originally developed by Kirigia et al*.* [23], and subsequently applied in the 2018 assessment of South Africa’s NHRS [22]. Thus, this study employed the following six steps suggested by Kirigia et al*.* [23]. The Excel Software developed by Microsoft (New York) was used in the data analysis.

#### Step 1: Delineate the goals and functions of NHRS

We applied the Pang et al. [9] NHRS framework that has two goals (advancing generation of R4H scientific knowledge and promoting its use for equitable health development) and four functions, including leadership and governance of NHRS; developing and sustaining NHRS resources; producing and utilizing R4H; and financing of NHRS.

#### Step 2: Selection of sub-functions for inclusion in Mauritius NHRS barometer

To facilitate comparison with 2014 and 2018 NHRS performance assessments [7, 23], the 17 sub-functions, listed in Table 1, were used in calculating the Mauritius NHRS Barometer.

**Table 1:** Sub-functions used in calculation of Mauritius NHRS barometer, 2020

Functions and sub-functions	Example to estimate a binary and continuous variable
**A. Leadership and Governance of R4H** 1. Existence of a national policy on research for health (R4H) *(binary variable)* 2. Existence of a Law / legislation relating to R4H *(binary variable)* 3. Existence of a strategic R4H plan *(binary variable)* 4. Existence of a national research ethics review committee *(binary variable)* 5. Existence of a national R4H prioritized agenda *(binary variable)* 6. Existence of a national health research focal point / unit *(binary variable)*	**The National R4H Policy Index (NR4HPI) for Mauritius NR4HPI** *The actual R4HP Score* is Mauritius is estimated being 0 since Mauritius does not have an R4H Policy. *Minimum R4HP Score* is the minimum possible value of 0, depicting the absence of an R4H policy. *Maximum R4HP Score* is the aspirational goalpost value of 1 depicting an R4H policy disseminated and implemented. The NR4HPI for Mauritius is, there equal to 0
**B. Developing and Sustaining Resources for R4H** 7. Existence of health research programme / directorate / department at Ministry of Health and Wellness (MOHW) *(binary variable)* 8. Number of technical & support staff in R4H programme per 100,000 population *(Continuous variable)* 9. Whether R4H programme has internet connectivity *(binary variable)* 10. Presence of a Medical Research and Innovation Council (MRIC) *(binary variable)* 11. Number of universities and colleges conducting R4H per a million population (*Continuous variable)* 12. Existence of non-governmental organization(s) undertaking R4H *(binary variable)*	**The Mauritius total number of universities and colleagues conducting R4H Index (UCR4HI)** *The Actual UR4H Score* of the sub-function on the total number of universities and colleges conducting R4H, was estimated to be 3.157 in step 4; the *Minimum UR4HN Score* would be 0 if Mauritius had no university conducting R4H; and *Maximum UR4HN Score* that any country can attain would be 5.39, that is the highest score in Africa (that of Cabo Verde). Thus UCR4HI = 0.586. (3.157 / 5.39)
**C. Producing and Utilizing R4H** 13. Existence of a national health research management forum (NHRMF) *(binary variable)* 14. Existence of knowledge translation platform (KTP) *(binary variable)* 15. Total number of R4H publications (between 1 December 2019 to 30 November 2020) per 100,000 population *(Continuous variable)*	***The Mauritius actual value of the sub-function ‘government allocation to R4H as a percentage of total MOHW budget in 2020/2021’ index (HRBI)*** *The Actual HRB Score* was estimated at 0.328 in step 4; the *Minimum HRB Score* is the least any government can allocate to R4H, which is 0; and regional and international target of allocating 2% of health sector annual budget to R4H was designated *Maximum HRB Score.* Thus, HRBI = 0.561 (0.328/2.0)
**D. Financing R4H** 16. Presence of funded R4H budget line within government budget *(binary variable)* 17. Percentage of MOHW budget allocated to R4H in 2020/2021 financial year *(Continuous variable)*	***The publication index (PUBI) for Mauritius*** In example 3 of Step 4, Mauritius *Actual PUB Score* was estimated to be 7.02; the *Minimum PUB Score* is 0, where a country has no peer-reviewed publications; and *Maximum PUB Score* of 12.514, that is, South Africa’s *Actual PUB Score* [49]. Thus HRBI = 0.561 (7.02/ 12.514)

Source: Adapted from Kirigia et al.[21]

#### Step 3: Collect data on each NHRS sub-function

Data was collected between August 2019 and November 2020. The study used ‘*EDCTP African Participating States: NHRS Assessment Questionnaire*’ [33]. It had ten sections, including health research policy; health research legislation; health research strategic plan; research regulation mechanism; health research programme; health research institute/council; national (public/private) universities; health research financing and budget; non-governmental organizations (NGOs) involved in health research; and actions needed to strengthen health research capacity. The questionnaire is an abridged version of the one used by Kirigia et al*.* in 2014 WHO African Region NHRS survey [15]. It was administered by one of the co-authors (YR) to the Ministry of Health and Wellness (MOHW) research programme, the Mauritius Institute of Health (MIH), Mauritius Research and Innovation Council (MRIC), University of Mauritius (UOM), the University of Technology Mauritius (UTM), and some NGOs, including PILS (Prévention Information et Lutte Contre le Sida), Cardiovascular Society of Mauritius and Mauritius Family Planning and Welfare Association.

The questionnaire survey was complemented with a review by three of the co-authors (AN, LM, and JMK) of pertinent information in various organizations websites, e.g. the Republic of Mauritius Government [34]; the MOHW [35]; the Ministry of Information Technology, Communication and Innovation [36]; the Ministry of Education, Tertiary Education, Science and Technology [37]; the Attorney General Office [38]; and public tertiary higher education institutions, including the UOM [39], the UTM [40], Université des Mascareignes (UdM) [41], the Open University of Mauritius (OUM) [42], the Mahatma Gandhi Institute [43], the Rabindranath Tagore Institute [44], the Mauritius Institute of Education (MIE) [45], the MIH [46], and the MRIC [47].

The total count of R4H publications in Mauritius was obtained from the 'PubMed' bibliographic database from 1 December 2019 to 30 November 2020 [48]. The search strategies have been uploaded by Musango and Kirigia [49] in the *Figshare* repository. The search for each of the 62 Medical Subject Headings (MeSH) yielded a total of 770 articles [49]. The authors (AN, LM, JMK) examined all the latter's abstracts and counted only those with at least one author having an affiliation / institutional address in Mauritius. The repeated/duplicated abstracts were omitted. This exercise reduced the total health sciences publication count to 89 articles for the period under consideration. Since part of the purpose of the study was to track progress in development of the Mauritius NHRS in producing R4H, it was necessary to count only articles published with at least one author having an affiliation / institutional address in Mauritius. Otherwise, publications by authors from foreign countries alone were deemed not to reflect capacity of Mauritius NHRS.

#### Step 4: Scoring of NHRS sub-functions

The study replicated the NHRS sub-function scoring methodology developed and applied in past studies in Africa [21, 23]. As indicated in Table 1, 13 of the 17 sub-functions were binary variables since they were about existence or non-existence of an NHRS sub-function Each of the binary sub-functions actual score a value of 1 if it existed and 0 if it did not. The remaining 4 sub-functions were continuous variables.

The actual scores for these four variables were calculated using the formulas below. First, the actual score for the number of universities and colleges conducting R4H (UR4H) was[21, 23]:

$$Actual UCR4H Score=\left({}^{UR4HN}\!\left/ \!{}_{Pop}\right.\right)\times \mathrm{1,000,000} population$$

where ‘*UCR4HN*’ is the total number of university colleges /schools of medicine conducting R4H, i.e. Anna Medical College and Research Centre (AMCRC), Sir Seewoosagur Ramgoolam Medical College (SSRMC), UOM, UTM; and ‘*Pop*’ is the total population of Mauritius in 2020. Thus, ‘UR4HN’ was 4 and ‘Pop’ was 1,267,000 [50].

Second the actual score value of the number of technical and support staff in the R4H programme (HRHR) was [21, 23]:

$$Actual R4HTS Score=\left({}^{HRHR}\!\left/ \!{}_{Pop}\right.\right)\times \mathrm{100,000} population$$

where ‘*HRHR*’ is the total number of technical and support staff in R4H programme/Unit, which includes two researchers and 19 administrative staff in the MIH [46], 15 staff in the NCD Health Promotion and Research Unit (NHPPR), and four staff in the Health Economics Unit (HEU) (from the NHRS survey questionnaire) [35]; and ‘Pop’ is the total population of Mauritius in 2020 [50]. Thus, since ‘*HRHR*’ = 40 and ‘Pop’ = 1,267,000 [50].

Third, the actual score value of R4H publications (*PUB)* in Mauritius between 1^st^ December 2019 and 30^th^ November 2020 was [21, 23]:

$$Actual PUB Score=\left({}^{TPUB}\!\left/ \!{}_{Pop}\right.\right)\times \mathrm{100,000} population$$

where ‘*TPUB*’ is the total number of R4H peer-reviewed publications (*PUB*) in Mauritius between 1^st^ December 2019 and 30.^th^ November 2020 from PubMed; and ‘*Pop*’ is the total population of Mauritius in 2020. Thus, the ‘T*PUB*’ was = 89 [49] and ‘*Pop*’ was = 1,267,000 [50]

Fourth, the actual value of the sub-function ‘government allocation to R4H as a percentage of total Ministry of Health and Wellness (MOHW) budget in Mauritius in 2020/2021 (HRB) was[21, 23]:

$$Actual HRB Score=\left({}^{R4HB}\!\left/ \!{}_{MOHWTB}\right.\right)\times 100\%$$

where ‘*R4HB*’ is government allocation to R4H in the financial year 2020/2021; ‘MOHW*TB*’ is the total MOHW budget in Mauritius in 2020/2021. The ‘*R4HB*’ was Rupees 38,395,000 and ‘*MOHWTB*’ was Rupees 11,700,000,000 [51, 52].

#### Step 5: Calculate NHRS Barometer sub-function indices for Mauritius

The following formula, which is like that used by the United Nations Development Programme (UNDP) in calculations of the Human Development Index [53], the Health Development Governance Index [54], Malawi NHRS Index [21], African NHRS Barometer [22, 23], and EDCTP African Participating States NHRS Barometer, was used to calculate all indices for the 17 individual sub-functions:

$$NHRSsub-function\;index=\left(\frac{Actual\;xi\;score-Minimum\;xi\;score}{Maximum\;xi\;score-Minimum\;xi\;score}\right)$$

where x_i_ is the i.^th^ sub-function, such as existence of a national research ethics review committee (NEC)

#### Step 6: Calculate the overall NHRS barometer score for Mauritius

After assessing the individual sub-function indices, Mauritius's overall NHRS barometer score was calculated as an arithmetic average of sub-functions indices '1' to '17*'.* Thus, the formula used in the overall NHRS barometer score (NHRS_BSCORE_) was as follows [21, 23]:

$${NHRS}_{BSCORE}=\left(\frac{\sum_{i=1}^{17}SFI}{{n}_{SF}}\right)={\theta }_{j} x 100\%.$$

SFI is the sub-function index; ‘n’ is the total number of NHRS sub-functions used in the NHRS barometer score calculations; $\sum_{i=1}^{17}SFI$ is the summation of NHRS sub-functions indices '1' to ‘*17*’; and theta symbol ${\theta }_{j}$ is the outcome of the operation $\left(\frac{\sum_{i=1}^{17}SFI}{{n}_{SF}}\right)$. The Mauritius ${NHRS}_{BSCORE}$ was computed on a scale of 0 (or 0%) to 1 (or 100%). A barometer score of 0% denotes that NHRS does not exist; 1% to 49% indicates that NHRS performance is below average; 50% suggests that NHRS performance is average; 51% to 99% shows that NHRS performance is above average; 100% implies that NHRS is performing optimally [21, 23].

## Results

Table 2 presents the ‘leadership and governance for R4H’, ‘developing and sustaining resources for R4H’, ‘producing and utilizing R4H’, and ‘financing R4H’ actual scores, maximum scores, minimum scores, sub-function NHRS indices, average NHRS individual function barometer scores, and the overall NHRS barometer score for Mauritius.

**Table 2:** Mauritius NHRS Barometer in 2020

**Sub-functions**	**Actual Score (A)**	**Maximum Score (B)**	**Minimum Score (C)**	**Sub-Function NHRS Index** **(D) = (A-C)/(B-C)**
**A. Leadership and Governance for R4H**				
1. Existence of a national R4H Policy	0	1	0	0
2. Existence of a legislation/law relating to R4H	1	1	0	1
3. Existence of a strategic R4H plan	0	1	0	0
4. Existence of a NEC	1	1	0	1
5. Existence of a national health research focal point/unit	1	1	0	1
6. Existence of a national R4H prioritized agenda	0	1	0	0
*** Average NHRS ‘leadership and governance’ barometer score***				***0.50 (50%)***
**B. Developing and Sustaining Resources for R4H**				
7. Existence of health Research Programme / directorate / unit (R4HP) at MOHW	1	1	0	1
8. Number of human resources for health research (HRHR) in R4HP per 100,000 population	3.157	100	0	0.0316
9. Whether R4HP has internet connectivity	1	1	0	1
10. Presence of a Mauritius Research and Innovation Council (MRIC)	1	1	0	1
11. Number of universities and colleges conducting R4H per a million population	3.157	5.39 (Cabo Verde)	0	0.586
12. Existence of NGOs undertaking R4H	1	1	0	1
*** Average NHRS ‘developing and sustaining resources’ barometer score***				***0.77 (77%)***
**C. Producing and Utilizing R4H**				
13. Existence of a NHRMF	0	1	0	0
14. Existence of a KTP	1	1	0	1
15. Total number of R4H publications (between 1 December 2019 and 30 November 2020) per 100,000 population	7.02	12.514 (South Africa)	0	0.561
*** Average NHRS ‘*** producing and utilizing R4H***’ barometer score***				***0.52 (52%)***
**D. Financing R4H**				
16. Presence of funded R4H budget line within government budget	1	1	0	1
17. Percentage of MOHW budget allocated to R4H in 2020/2021 financial year	0.328	2.0 (Regional target [3])	0	0.164
*** Average NHRS ‘*financing R4H*’ barometer score***				***0.582 (58.2%)***
** Overall NHRS barometer score ((10.3426 sum of sub-function indices divided by 17) × 100%)**				**60.84%**

Note: Cabo Verde and South Africa had respectively the highest barometer scores for ‘developing and sustaining resources for R4H’ and ‘producing and utilizing R4H in the African Region during the 2018 survey. Thus, they were used as the benchmarks (maximum) for the two NHRS functions

### Leadership and governance of R4H

Table 2 section ‘A ‘shows the Mauritius NHRS ‘leadership and governance of R4H’ six sub-function indices and the average leadership and governance function barometer score in 2020. First, the country had no dedicated national R4H policy [55], and thus, this sub-function had an NHRS index of 0%.

Second, Mauritius has a legal framework that underpins the leadership and governance of R4H, leading to a sub-function index of 1. The Protection of Human Rights Act of 1999 provides for establishing a National Human Rights Commission mandated, among others, to promote and protect human rights, and harmonize national legislation and practices with international human rights instruments [56]. Article 3(a) of the Mauritius Constitution affirms “the right of the individual to life, liberty, security of the person and the protection of the law” [57]. Although the Constitution does not have provision for the right to health care, the Public Health Act of 1925 [58] regulates practices to prevent morbidity and mortality due to communicable diseases and ensuring an environment free of health hazards.

There exist laws that regulate and control the conduct of various groups of the health workforce, including the Allied Health Professionals Council, the Dental Council, the Medical Council, the Nursing Council, the Pharmacy Council, and the Veterinary Council [59, 60].

The Clinical Trials Act 8 of 2011 established (a) the Clinical Research Regulatory Council (CRRC); (b) the National Ethics Committee (NEC); and (c) the Pharmacovigilance Committee (PC) [61]. The CRRC is legally mandated to licence clinical trials; examine and approve qualifications of investigators; consider reports of the NEC, PC and the Trade and Therapeutics Committee (TTC); issue guidelines for the safe and ethical conduct of clinical trials; maintain a register of clinical trials and related publications; assure safety and protection of research subjects health and welfare (Article 4).

The MRIC performs leadership and governance of the NHRS complemented by the CRRC, NEC, PC, and institutional research ethics committees [62, 63]. The MRIC Act of 2019 empowers the Council to develop, operationalise and coordinate a national strategy for research, development, and innovation, as well as facilitate collaboration, exchange of knowledge between researches and funding partners [63]. The MRIC is an autonomous corporate body under the Ministry of Information Technology, Communication, and Innovation. However, there is no memorandum of understanding between the MOHW and the MRIC.

Third, the country did not have a dedicated national R4H strategic plan leading to an NHRS sub-function index of 0%. However, the Mauritius health sector strategic plan (HSSP) of 2020–2024 provides for implementation of health research strategies to bridge the existing gaps in NHRS leadership and governance [55]. The HSSP strategic goal 19 aims caters for actions related to leadership and governance of R4H,including the development of a prioritized R4H agenda, an NHRP and strategy; strengthening of the capacities of the NEC and institutional research ethics review committees; institutionalizing knowledge translation platforms; development of a roadmap to promote Mauritius into a medical research hub and as a destination for clinical trials [64]. Also, the MRIC [47], UOM [39], and UTM [40] have research strategies that include health sciences.

Fourth, existence of a functional NEC within the MOHW resulted in an NHRS sub-function index of 1 (or 100%). According to the Clinical Trials Act 8 of 2011 Article 8, the NEC is mandated primarily to advice MRIC on protection of clinical trials subjects, ensure respect of international ethical and scientific standards, prepare guidelines, and archive records of proceedings relating to clinical trials [64]. The NEC is complemented by institutional ethics committees, e.g., the Institutional Ethical and Review Board of SSRMC [64], and the UOM Research Ethics Committee [39].

Fifth, there are three National R4H Focal Points in Mauritius, leading to an NHRS sub-function index of 1 (or 100%). They are the Lead Health Analyst at the MOHW [35], the Executive Director of the MIH [46], and the Executive Director of the MRIC [47].

Sixth, the country did not have a national prioritized R4H agenda, which resulted in a sub-function NHRS index of 0 (or 100%). The average leadership and governance function score was 0.50 (or 50%), signifying an average performance.

Extant challenges for ‘leadership and governance of NHRS’ include lack of national R4H policy, strategic plan, and prioritized agenda; lack of memorandum of understanding between MOHW and some of the national universities, colleges and institutes in Mauritius conducting R4H; lack of national guidelines on the development of collaborative agreements on R4H involving Mauritius institutions and those outside the country; sub-optimal inter-sectoral research coordination; insufficient coordination between the national R4H focal points in MRIC, MIH, and MOHW; and need to strengthen the capacity of research ethics committees.

### Developing and sustaining R4H resources

Table 2 section ‘B’ presents the Mauritius NHRS ‘developing and sustaining R4H resources’ six sub-function indices and the average ‘developing and sustaining resources’ function barometer score in 2020.

First, the country had a R4HP under the aegis of the MOHW, thus meriting a sub-function NHRS index of 1 (or 100%). The R4HP consists of the MIH, HEU, and NHPPR. The MIH, established by the MIH Act 36 of 1989, has the onus to carry out health systems research whilst functioning as a resource centre for the production, exchange and promotion of health learning and health information material [46].

Second, the R4HP had a total of 40 staff (HRHR), including two full-time and 19 part-time researchers in the MIH, four full-time staff in the HEU, and 15 research staff in the NHPPR. Since ‘*HRHR*’ was 40 and ‘Pop’ was 1,267,000 [46], the actual HRHR score was 3.157, i.e., 40 staff divided by 1,267,000 people times 100,000 population. The NHRS sub-function of HRHR staff per 100,000 population index was 0.0316 (or 3.16%), that is, (actual HRHR score of 3.157 – minimum score of 0) divided by (maximum HRHR score of 100 – minimum HRHR score of 0).

Third, each of the R4HP staff had a computer connected to email and the internet services, resulting in an NHRS internet connectivity sub-function score of 1 (or 100%). The R4HP do not have laboratories and capacity (human or physical) to undertake clinical trials.

Fourth, the existence of the MRIC yielded an NHRS sub-function index of 1 (or 100%). The MRIC has 23 research staff and 31 administrative staff but who are not all dedicated to R4H. During the study period, the MRIC has completed eight health-related projects in-house and had funded other institutions in Mauritius (e.g., SSRMC, UOM) to undertake 12 health-related projects [47, 65]. However, the MRIC has no laboratories and human (or physical) capacity to conduct clinical trials.

Fifth, there were four universities/colleges that conduct R4H in Mauritius (UR4HN), including the AMCRC, the SSRMC, the UOM, and the UTM. Given that ‘UR4HN’ was 4 and ‘Pop’ was 1,267,000 [50], the *Actual UR4H* score was 3.157, i.e., UR4HN of four divided by Pop of 1,267,000 multiplied by one million population. The NHRS sub-function of the number of universities and colleges conducting R4H per a million-population index was 0.586 (or 58.6%), that is, (actual UR4HN score of 3.157 – minimum UR4HN score of 0) divided by (maximum UR4H score of 5.39 – minimum UR4HN score of 0). Besides there are some other governmental and inter-governmental entities that undertake R4H, including four public hospitals, the Medical Council of Mauritius [66], the Indian Ocean Commission Health Monitoring Unit [67], the MIE, and the Ministry of Agro-Industry and Food Security.

Finally, the country has non-government organizations (NGO), e.g., Prévention Information et Lutte Contre le Sida (PILS) [68], and Mauritius Family Planning and Welfare Association [69]. Also, some private companies exist which conduct health-related research, e.g., the Clinglobal Limited [70], CIDP Mauritius [71], CAP Research [72], and Clinear Research [73]. Furthermore, health development partners, such as the Mauritius WHO Country Office also conduct and support R4H. Therefore, the NHRS sub-function of NGOs undertaking R4H had an index of 1 (or 100%).

The average barometer score for NHRS function of ‘developing and sustaining resources’ was 0.77 (or 77%), denoting above-average performance. The function had a performance gap of 23%. Thus, to optimize the NHRS function of developing and sustaining resources for R4H, there is a need to increase the density of institutions with requisite human and infrastructural capacities for conducting R4H. The NHRS function of ‘developing and sustaining resources’ is constrained by insufficient human resources for health research, and lack of laboratory capacity at MRIC and MIH to conduct clinical trials.

### Producing and utilizing R4H

Table 2 section ‘C’ depicts the average barometer score for the Mauritius NHRS ‘producing and utilizing R4H’ function and indices for the three sub-functions of ‘producing and utilizing R4H’ in 2020. First, the country does not have a national health research management forum (NHRMF), that is, an organ representing all key stakeholders and the MOHW as its Secretariat. Among other responsibilities, if the NHRMF existed, it would have facilitated dissemination and utilisation of research results and advocacy for R4H. Thus, the NHRS sub-function of NHRMF has an index of 0%.

Second, a knowledge translation platform (KTP) exists in Mauritius that collates, translates, synthesizes, and communicates research to inform health policy and practice. Given NHPPR and HEU's existence within the MOHW, which currently play the KTP role, the NHRS function of KTP had an index of 1 (or 100%). For example, the collaboration of the NHPPR and HEU with international institutions and academia to convene conferences to promote research, and knowledge sharing in areas of NCDs and health financing [74–76].

The HRP (consisting of NHPPR and HEU) conducts R4H. For instance, the NHPPR undertook various NCD-related studies and surveys [77, 78]. The HEU produced National Health Accounts [79, 80]. National AIDS Spending Assessment [81, 82], and Hospital Cost Centre Projects. The results were used to re-prioritize strategies concerning NCDs prevention, health promotion and curative care [51].

Lastly, the total number of R4H journal articles for Mauritius between 1 December 2019 and 30 November 2020 was 7.02 per 100,000 population and, thus, resulted in a NHRS R4H publication sub-function index of 0.561 (or 56.1%), which was above average, but below optimal performance by 43.9%.

The barometer score for the NHRS function of ‘producing and utilizing research’ was 0.52 (or 52%), implying a performance above average. Despite that, the function performance was suboptimal by 48%.

The main factors constraining NHRS ‘producing and utilizing R4H’ function include the need for optimal use of research findings; increased demand for research; enhanced research culture among health care providers; improved flow of information between NGOs and the relevant MOHW departments in the course of ongoing research; and establishment of a NHRMF to facilitate dissemination of research findings, promote uptake of research in policy, product development and innovation, and collaboration between researchers, innovators and funding partners.

### Financing R4H

Table 2 section ‘D’ presents the barometer score for the Mauritius NHRS function of ‘financing R4H’ in 2020. The R4H financing—in order of importance—is from government tax revenues, private sector companies, multilateral and bilateral donors, international NGOs, and local NGOs.

The function of financing R4H has two sub-functions. First, since health research budget exists within the government budget, the NHRS sub-function index was 1 (or 100%). In 2020/2021 financial year, the government’s overall budget was Rupees (Rs) 185.03 billion; of which Rs 11.7 billion was allocated to the MOHW [51]. Out of the latter, some Rs36.595 million was allotted R4H activities, including those implemented by MIH [51]. The Ministry of Information Technology, Communication and Innovation allocated to MRIC Rs45 million for research activities related to 25 ministries. This represented an amount of Rs1.8 million of the MRIC for R4H [51, 52]. Therefore, we estimate that approximately Rs38.395 million is for R4H, which is an underestimated value as universities and colleges' spending was not available.

Second, the government allocation to R4H as a proportion of MOHW budget in 2020/2021 financial year was 0.328, that is, Rs 38.395 million divided by Rs 11.7 billion times 100%. Since the target recommended by the Commission on Health Research for Development [8] and WHO RC [5] is to allocate at least 2% of the health budget on R4H, the sub-function index equals 0.164 (or 16.4%), that is, 0.328 divided by 2.0.

The average Mauritius NHRS ‘financing research for health’ function barometer score was 0.582 (or 58.2%), above average. However, there was a deficit of 41.8% in the function performance. The NHRS function of ‘financing R4H’ is constrained by lack of evidence on total national spending on R4H by all government sectors, private-for-profit sector, private-not-for-profit sector, inter-governmental organizations, external collaborating institutions, and health development partners; disruption of some research projects undertaken by NGOs due to insufficient funding; and inadequate government funding R4H.

### The overall NHRS barometer score for Mauritius

Table 2 summarizes Mauritius NHRS barometer score for 2020. Four of the 17 sub-functions had a zero index, signifying absence R4H policy, R4H strategic plan, prioritized R4H agenda, and a NHRMF. One sub-function scored less than 10%, that is, HRHR in R4HP per 100,000 population, while one other scored above 10% but below 50%, that is, Government budget allocation to R4H as a proportion of MOHW budget in 2020/2021 financial year. Two sub-functions scored between 50 and 59%, that is, densities of universities and colleges conducting R4H, the number of R4H journal articles published in a year; and the remaining nine sub-functions scored 100% (optimal).

The four NHRS functions average barometer scores were 50.0% for ‘leadership and governance for R4H’, 77.0% for ‘developing and sustaining resources for R4H’, 52.0% for ‘producing and utilizing R4H’, and 58.2% ‘financing R4H’.

Given the 17 sub-function indices contained in Table 2 above, Mauritius overall barometer score ( ${(NHRS}_{BSCORE})$ was calculated as follows:

$${\varvec{N}}{\varvec{H}}{\varvec{R}}{{\varvec{S}}}_{{\varvec{B}}{\varvec{S}}{\varvec{c}}{\varvec{o}}{\varvec{r}}{\varvec{e}}}=\left(\frac{\sum_{{\varvec{i}}=1}^{17}{\varvec{S}}{\varvec{F}}{\varvec{I}}}{{{\varvec{n}}}_{{\varvec{S}}{\varvec{F}}}}\right)=\left(\frac{10.3426}{17\boldsymbol{ }}\right)=0.6084\boldsymbol{ }{\varvec{x}}\boldsymbol{ }100\boldsymbol{\%}=60.84\boldsymbol{\%}.$$

where $\sum_{{\varvec{i}}=1}^{17}{\varvec{S}}{\varvec{F}}{\varvec{I}}$ (sum of 17 NHRS sub-function indices) equals 10.3426, and ${{\varvec{n}}}_{{\varvec{S}}{\varvec{F}}}$ (number of NHRS sub-functions) is 17. The Mauritius ${NHRS}_{BSCORE}$ of 60.84% denotes above-average NHRS performance. Therefore, although Mauritius's overall NHRS barometer score was above average, it fell short of optimal performance by 39.16%.

## Discussions

### Summary of key findings

- 9 (52.94%) of the 17 NHRS sub-functions had indices of 100%, implying the existence of R4H legislation(s), functional NEC, national R4H focal point(s), R4H programme, internet connectivity in the R4H programme, MRIC, NGOs undertaking R4H, KTP, and an R4H budget line within the government budget.
- The NHRS functions of leadership and governance, developing and sustaining resources, producing and utilizing R4H, and financing R4H had barometer scores of 50% and above.
- The overall NHRS barometer score for Mauritius in 2020 of 60.84% was above average, but short of optimal performance by 39.16%.

### Mauritius temporal NHRS barometer scores comparison

**Fig. 1:**
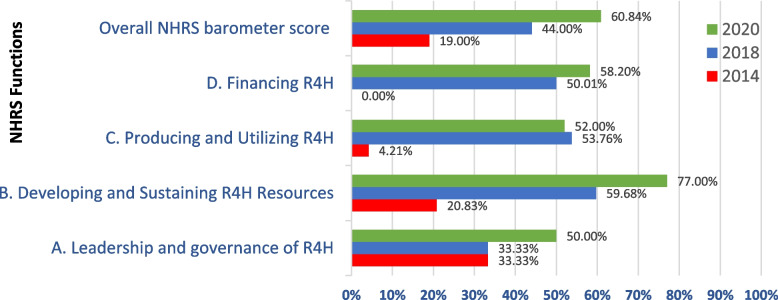
Comparison of Mauritius NHRS Barometer Scores (percentage) for 2014, 2018 and 2020, %

Figure 1 demonstrates that between 2018 and 2020, the overall NHRS barometer score improved by 38.27%, i.e., from 44.0% to 60.84%. The improvements are attributed to performance growth of 50.0% in leadership and governance, 29.0% in developing and sustaining resources, and 16.4% in financing R4H.

### Leadership and governance of R4H

In Mauritius, the NHRS leadership and governance function performance improved by 50.0%, that is, from 33.3% in 2014 to 50.0% in 2020. The improvement is attributed to the existence of R4H laws, functional NEC, and active national focal points. As mentioned in the results section, there exist three national R4H points in the MOHW, MIH, and MRIC, and hence, there might be a need to improve communication and coordination between the trio. To optimize the leadership and governance function, as envisaged in the HSSP 2020–2024, the country should develop a national R4H policy [15, 83, 84], strategic plan [85, 86] and prioritized agenda [55].

A national R4H agenda (NR4HA) is essential as it provides guidance to all stakeholders regarding priority research needs, improves efficiency in using research resources, and attracts more investments from all sources. The process of developing a research agenda is contained in Kirigia et al. [32] and the R4H priority setting guidelines by the Council on Health Research for Development (COHRED) [87] and the Global Forum for Health Research [88].

To effectively perform its NHRS leadership and governance function [89], the MRIC ought to spearhead the development of a national R4H policy, strategic plan, and a prioritized agenda. The three would be invaluable reference documents for all organizations, institutions, and persons involved in the conduct and use of R4H.

Furthermore, MRIC ought to consider developing national guidelines on establishing formal collaboration agreements between national and foreign R4H institutions to facilitate intellectual property management [90]. The EU guidelines underscore the main factors that need to be considered before entering a collaboration with a non-Mauritius entity, including a risk–benefit analysis, scope and objective, maintenance of confidentiality, assessment of intellectual property legal framework, and obligations.

### Developing and Sustaining R4H Resources

The performance of NHRS ‘developing and sustaining resources’ function enhanced from 20.83% in 2014 to 77%, that is, 269.66% increase. The growth in performance could be attributed to the existence of a research programme (consisting of NHPPU and HEU within the MOHW) that are computerized and connected to the internet to facilitate research; the presence of the MRIC that conducts and funds R4H besides its functions of coordination; and engagement of non-governmental organizations undertaking R4H. The deficits in performance were in numbers of HRHR in the research programme and the number of universities and colleges conducting R4H.

Some actions can be taken to increase HRHR capacities. First, leverage the Pan African University for the training of Doctor of Philosophy (PhD) level health-related disciplines researchers [91]. Second, tap into the Special Programme for Research and Training in Tropical Diseases (TDR) postgraduate training fellowships [92] that are offered through three regional centres, including the University of Ghana, School of Public Health, Ghana [93]; the University of the Witwatersrand, School of Public Health, South Africa [94]; and the University of Zambia, Department of Public Health, Zambia [95].

Third, even though the Mauritian public higher education sector has six public higher education institutions and three specialised public institutes, only the UOM, UTM, MIH and MIE have evidence of conducting R4H. These are potentially significant underutilised research resources in the country. Thus, there is a need to boost R4H human and infrastructural capacities in UdM and OUM.

Fourth, some of the higher learning institutions in Mauritius should explore the possibility of applying for the CARTA (Consortium for Advanced Research Training in Africa) membership to boost multidisciplinary R4H capacities [96]. CARTA is a collaborative consortium established in 2008, jointly led by the African Population and Health Research Centre based in Kenya, and the University of the Witwatersrand, South Africa. Its mission is to build high-level capacity for population and public health-related research in African universities by strengthening R4H infrastructure and governance capacities and the support of junior faculty members to undertake their population and public health doctoral training in local collaborative programmes [94, 95, 97].

### Producing and utilizing R4H

The performance of NHRS producing and utilizing research function grew from 4.214% in 2014 to 52.0% in 2020, i.e., 1,133.98% increase. However, compared to the function mean barometer score of 53.758% in 2018, there was a slight decrease in 2020 of 3.27%. Mauritius might attain a maximum barometer score for producing and utilizing R4H through action in two areas.

First, the country could consider establishing a functional NHRMF within the MRIC that would serve as a platform for exchange between researchers and health policymakers. An NHRMF is an organ representing all key R4H stakeholders with the focal government entity as its Secretariat, i.e., MRIC in Mauritius. Even though Mauritius does not currently have a dedicated NHRMF, the MRIC is legally mandated to perform most of the roles and responsibilities of an NHRMF [47]. As done in some countries such as Ghana [98], a two-day NHRMF symposium could be convened every two years, to give human resources for R4H an opportunity to showcase methodologies and findings from studies conducted in Mauritius. Authors could then be encouraged to polish their manuscripts for publishing in special issues of peer-reviewed journals for wide dissemination.

Second, undertake multi-faceted actions to boost the number of R4H articles authored or co-authored by research institutions in Mauritius. Such actions might include: (a) establishment of formal collaboration between national and international R4H institutions; (b) UOM, UOT, or SSRMC may consider applying for WHO Collaborating Centre status to participate in collaborative R4H developed under the Organization’s leadership in areas of comparative advantage, e.g. NCDs [99]; (c) UOM, UOT, or SSRMC may also consider formal collaboration with the Africa Centres for Disease Control And Prevention (Africa CDC) in chronic diseases [100]; (d) MRIC could negotiate with national research institutions to make publication of articles in peer journals a critical criteria for annual salary increments and for recruitment and career progression; (e) MRIC could also acknowledge researchers with most pertinent articles in peer reviewed journals; (f) MRIC could advocate with the Ministry of Higher Education to include number of peer reviewed journal articles in the annual performance contracts for universities and colleges [101]; (g) MRIC could develop a roadmap to promote the country into a medical research hub.

A comparison of the total number of R4H journal articles for Mauritius between 1 December 2019 and 30 November 2020 with South Africa revealed that the latter score was 12.514 (highest in the WHO African Region) against 7.02 per 100,000 population for Mauritius [55].

### Financing R4H

NHRS financing function's performance improved from 50.013% in 2018 to 58.2% in 2020, i.e., a 16.37% increase. The Mauritius spending on R4H of approximately 0.328% of the health sector budget is below the regional [5] and international [8] target of 2%. The non-achievement of the target could be attributed to two factors.

First, in 2018, about 10% of the general government expenditure was spent on health [102] which was lower than the 15% target set by the Heads of State and Government of the Organization of African Union (OAU) in 2001 [103]. Two, the domestic general government health expenditure (GGHE) of 3% of GDP in 2018 was lower than the "target of government spending on health of at least 5% of GDP for progressing towards Universal Health Coverage" recommended by McIntyre, Meheus and Røttingen [104].

MRIC ought to advocate with the MOHW to allocate at least 2% of the health sector budget on R4H, in line with WHO Regional Committee Africa resolution [5]. Also, the MRIC ought to embark on evidence-based advocacy with the Ministry of Finance to establish a levy to finance National Research and Innovation Fund (NRIF) [47]. To stand a good chance of success, the MRIC will need to use evidence on the linkages between research and economic development when couching its advocacy messages with the Ministry of Finance. For instance, local evidence of returns to Mauritius publicly funded R4H regarding the net value of improved health status would be pivotal [105–107].

### Limitations of the study

The study reported in this paper had some limitations. First, the estimated government spending on R4H was underestimated because the universities and colleges spending was not available. Second, due to budgetary constraints, the total number of R4H articles published by one author having an affiliation / institutional address in Mauritius were obtained from a search of only PubMed. Thus, there is some possibility that some publications may have been missed.

A caveat when interpreting the result is that the 2018 assessment reported in Rusakaniko et al. [7] did not include the number of articles published in peer-reviewed journals. The 2014 baseline NHRS calculations included the number of articles published in peer-reviewed journals obtained from Uthman et al. [108].

## Conclusion

The study succeeded in estimating NHRS barometer score for Mauritius in 2020 and comparing it to the 2018 and 2014 scores; identifying the gaps in NHRS performance; recommending possible courses of action for boosting the performance of the Mauritius NHRS. Between 2014 and 2020, the overall NHRS barometer score improved by three-fold, i.e., from 19% to 60.84%.

It will be necessary to bridge the existing gap in overall NHRS barometer score of 39.16% to sustain the production of evidence needed to guide Mauritius towards attaining the SDG3 goal on ‘ensuring healthy lives and promoting well-being for all residents at all ages’ [109].

That will entail:

- 1.Formulation and implementation of a national R4H policy, strategic plan, and a prioritised agenda [55].
- 2.Planned development of a critical mass of human resources for health research [110].
- 3.Establishment of an NHRMF within MRIC to promote production and use of evidence in health policy, planning, and decision-making to further accelerate the achievement of SDG3 [15].
- 4.Conduct of a national R4H accounts to accurately estimate the current total expenditure on health-related research [111].
- 5.Evidence-based advocacy with the MOHW to allocate at least 2% of the health sector budget for strengthening NHRS [5, 8].

As the country endeavours to strengthen its health system and progress towards UHC, there still abounds concerns about the appropriateness of service delivery models and whether they are responsive to peoples’ needs. Against this background, R4H will undoubtedly have a critical role in terms of designing affordable service delivery models and identifying strategies to optimise the use of existing solutions to address health systems challenges hampering attainment of SDG Target 3.8 of UHC.

## Data Availability

Underlying data A Figshare: The Mauritius PubMed Search Strategy [1 December 2019 to 30 November 2020]. https://doi.org/10.6084/m9.figshare.13611767. B The data used in estimating the Mauritius NHRS Barometer in 2020 can be found in Table 2 of the manuscript.

## Abbreviations

CARTA: Consortium for advanced research and training in Africa
CMNND: Communicable, maternal, neonatal, and nutritional diseases
COHRED: Council on health research and development
CRRC: Clinical research regulatory council
EDCTP: European and developing countries clinical trials programme
GDP: Gross domestic product
GGHE: General government health expenditure
HEU: Health economics unit
HRHR: Human resources for health research
HSSP: Health sector strategic plan
KTP: Knowledge translation platform
MIE: Mauritius institute of education
MIH: Mauritius institute of health
MOHW: Ministry of health and wellness
MRIC: Mauritius research and innovation council
NCD: Noncommunicable diseases
NEC: National ethics committee
NGO: Nongovernmental organization
NHPPR: NCD, health promotion and research unit
NHRMF: National health research management forum
NHRS: National health research system
NR4HA: National research for health agenda
NRIF: National research and innovation fund
OAU: Organization of African unity
OdM: Universite des mascareignes
OOPS: Out of pocket payments
OUM: Open university of mauritius
PC: Pharmacovigilance committee
RC: WHO regional committee for Africa
R4H: Research for health
R4HP: Research for health programme
SDG: Sustainable development goal
SSRMC: Sir Seewoosagur Ramgoolam Medical College
TDR: Special programme for research and training in tropical diseases
TTC: Trade and therapeutics committee
UdM: Université des mascareignes
UHC: Universal health coverage
UHC SCI: UHC service coverage index
UNDP: United nations development programme
UOM: University of Mauritius
UTM: University of technology, Mauritius
WHO: World Health Organization
